# Abnormal Stability of Dynamic Functional Architecture in Amyotrophic Lateral Sclerosis: A Preliminary Resting-State fMRI Study

**DOI:** 10.3389/fneur.2021.744688

**Published:** 2021-10-13

**Authors:** Jin Wei, Jia-Hui Lin, Li-Min Cai, Jia-Yan Shi, Xiao-Hong Zhang, Zhang-Yu Zou, Hua-Jun Chen

**Affiliations:** ^1^Department of Radiology, Fujian Medical University Union Hospital, Fuzhou, China; ^2^Department of Neurology, Fujian Medical University Union Hospital, Fuzhou, China

**Keywords:** amyotrophic lateral sclerosis, dynamic, functional connectivity, functional stability, functional magnetic resonance imaging

## Abstract

**Purpose:** Static and dynamic analyses for identifying functional connectivity (FC) have demonstrated brain dysfunctions in amyotrophic lateral sclerosis (ALS). However, few studies on the stability of dynamic FC have been conducted among ALS patients. This study explored the change of functional stability in ALS and how it correlates with disease severity.

**Methods:** We gathered resting-state functional magnetic resonance data from 20 patients with ALS and 22 healthy controls (HCs). The disease severity was assessed with the Revised ALS Functional Rating Scale (ALSFRS-R). We used a sliding window correlation approach to identify dynamic FC and measured the concordance of dynamic FC over time to obtain the functional stability of each voxel. We assessed the between-group difference in functional stability by voxel-wise two-sample *t-*test. The correlation between the functional stability index and ALSFRS-R in ALS patients was evaluated using Spearman's correlation analysis.

**Results:** Compared with the HC group, the ALS group had significantly increased functional stability in the left pre-central and post-central gyrus and right temporal pole while decreased functional stability in the right middle and inferior frontal gyrus. The results revealed a significant correlation between ALSFRS-R and the mean functional stability in the right temporal pole (*r* = −0.452 and *P* = 0.046) in the ALS patients.

**Conclusions:** ALS patients have abnormal stability of brain functional architecture, which is associated with the severity of the disease.

## Introduction

Amyotrophic lateral sclerosis (ALS) is an adult-onset neurodegenerative motor neuron disorder. Progressive upper and lower motor neuron involvement occurs during the course of the disease (1–3). ALS is considered to be a multisystem disorder with substantial extra-motor involvement, such as in the cognitive system and behavioral system; however, the etiology of ALS remains unclear (2–4). The course of ALS progresses rapidly, and most patients suffer respiratory failure and die within 3–5 years of the onset of symptoms (2, 4). There is still no effective treatment for ALS, and timely diagnosis of ALS is the key to early intervention and the improvement of the prognosis (5). Hence, it is important to find new neuroimaging signs that can reveal the pathogenesis of ALS and monitor the disease progress.

Resting-state functional magnetic resonance imaging (fMRI), which is based on blood oxygen level-dependent effects, has been widely used to record spontaneous brain activity signals (6). Because of the ease of application of resting-state fMRI and its ability to characterize complex brain circuits, it has been used in many neuroimaging studies (7). Functional connectivity (FC), which is measured based on resting-state fMRI signals, has shown promise as a potential biomarker for its association with neurological disorders (8). For example, the presence of the widespread reorganization of FC has been documented in ALS patients and reveals the potential severing as a biomarker for monitoring disease development (8). By measuring the static FC (the index reflecting the averaged strength of functional coordination among distinct brain regions), resting-state fMRI studies have found significantly reduced FC in sensorimotor networks (SMNs) and brain networks related to cognition and behavior in ALS (9–11). The FC alterations in ALS are in keeping with the change of structural connections in the motor system and extra-motor systems (12). In addition, a recent study has revealed the alteration of dynamic FC properties in ALS, which is correlative with the severity of motor dysfunction (13). Taken together, both static and dynamic FC analyses hold great promise to explain the mechanisms underlying ALS.

In a wide range of task states and unrestricted resting states, the brain is always in an active state and the neural signals are spontaneous and highly dynamic (14, 15). Complex cognitive and motor functions necessitate the brain's coordination of information from multiple modes over time (16, 17). Although the functional architecture of the brain is dynamic in nature, its functional stability in a continuous state is critical for maintaining normal brain functions (18). For example, the property of functional stability is an important feature of consciousness. For conscious processing to occur, the stable and reproducible representation of high-quality information by a distributed activity pattern in the higher cortical areas is critical (19). In addition, the nervous system requires functional stability in order to integrate information (19) and maintain cognitive acuity (20). In addition, the stability of the activation pattern in the primary motor cortex is thought to be associated with motor function (21, 22).

Currently, the functional stability property of the brain has attracted increasing attention (18). A recent study conducted a voxel-wise analysis of resting-state fMRI data and characterized the stability of the brain's functional architecture by assessing the concordance of dynamical functional connections over time (18). The results indicated that high stability was found in high-order association regions, while unimodal regions exhibited lower stability during resting-state scans (18). The higher the stability value, the more concordant and stable the dynamic functional architecture configuration is over time, indicating the maturity of the FC mode of high-order association regions. The lower the stability value, the lower the capacity is to coordinate information over time, indicating that it can frequently and quickly transfer from one brain state to another. Indeed, the connections between the unimodal regions and other brain regions change constantly with different tasks or states so that the brain can adapt to different timescales (23). Of note, a recent resting-state fMRI study has shown that the analysis of functional stability contributes to exploring the mechanisms underlying various neuropsychiatric disorders (24).

Herein, we conducted the first analysis of functional stability based on resting-state fMRI data; and we aimed to contribute new understandings of the mechanism about functional stability in ALS and investigate its correlation with disease severity.

## Methods

### Subjects

The subjects included in this investigation comprised 20 patients diagnosed with sporadic ALS and 22 healthy controls (HCs). We used the El Escorial criteria (25) for the diagnosis of ALS, and implemented the revised ALS Functional Rating Scale (ALSFRS-R) to assess the severity of the disease. The lower ALSFRS-R score indicated the increased severity of disease. Of the patients, 5 ones were taking Riluzole when they were recruited. The age, sex, and education levels of the subjects in the two groups were comparable (Table 1). We used the following exclusion criteria: (1) other neuropsychiatric disorders, including Alzheimer's disease, Parkinson's disease, epilepsy, or depression; (2) patients undergoing treatment with psychotropic medications; (3) patients who developed respiratory failure or other serious disorders, including heart failure or cancer; and (4) patients with contraindication of MRI examination. The local Research Ethics Committee approved this study, and all study participants gave written informed consent.

**Table 1:** Demographic and clinical data of the study participants.

	**Healthy controls (*n* = 22)**	**ALS patients (*n* = 20)**	***P*** **-value**
Age (years)	56.4 ± 4.9	57.2 ± 4.7	0.45
Sex (females/males)	8/14	6/14	0.66
Education (years)	8.1 ± 3.0	7.1 ± 3.0	0.32
Site of onset (bulbar/cervical/lumbosacral)	–	2/12/6	–
Diagnostic category (definite/probable/possible)	–	6/7/7	–
ALSFRS-R score	–	39.5 ± 6.8	–
Disease duration (months)	–	16.2 ± 13.8	–
Disease progression rate	–	0.77 ± 0.60	–

*ALS, amyotrophic lateral sclerosis; ALSFRS-R, revised ALS Functional Rating Scale. The following equation was used to estimate the rate of disease progression: (48–ALSFRS-R)/Disease duration. “–” indicates that no data are available*.

### MRI Data Acquisition

The MRI data were acquired using a 3.0T scanner (Prisma, Siemens Medical Systems, Erlangen, Germany). Resting-state functional images were captured with the multiband slice acquisition method using the echo-planar imaging sequence, and the parameters were as follows: multiband factor = 4, TR = 500 ms, TE = 30 ms, matrix = 76 × 76, flip angle = 50°, FOV = 228 × 228 mm, slice thickness = 4.5 mm (without interslice gap), 32 axial slices, and 800 volumes. All participants were instructed to keep their eyes closed, think of nothing in particular, and remain still. In order to carry out spatial normalization of functional images, we collected three-dimensional T1-weighted images (resolution = 1 mm^3^) with the magnetization-prepared rapid gradient-echo (MPRAGE) sequence.

### Functional MRI Data Pre-processing

The functional MRI data pre-processing was conducted using Statistical Parametric Mapping software (SPM 12, http://www.fil.ion.ucl.ac.uk/spm) as well as the Data Processing and Analysis of Brain Imaging toolbox (DPABI, Version6.0, http://rfmri.org/DPABI). The initial 40 volumes were excluded while the signal reached equilibrium and the participants adapted to the scanning noise. Realignment was carried out to correct the motion between time points. The head motion parameters were calculated by estimating the translation in each direction and the angular rotation on each axis for each volume. In addition, the frame-wise displacement (FD), which indexes the volume-to-volume changes in head position, was calculated. None of the participants had a range of movement >3 mm translation or 3 degrees of rotation, and all fMRI data included in the final sample were within the defined motion thresholds of mean FD lesser than 0.15 mm. Nuisance covariates, which included the linear trend, the estimated motion parameters based on the Friston-24 model, the white matter signal, and the cerebrospinal fluid signal, were regressed out from the functional signal. During normalization, the individual structural images were co-registered with the mean functional image. The transformed structural images were then segmented and normalized to the Montreal Neurological Institute (MNI) space through the use of a high-level non-linear warping algorithm, that is, the Diffeomorphic Anatomical Registration Through Exponentiated Lie algebra (DARTEL) method (26). Then, the deformation parameters that were estimated in the aforementioned step were used to spatially normalize each functional volume to MNI space, and the functional volumes were resampled into a 3-mm cubic voxel. The datasets were subsequently band-pass temporal filtered (0.01–0.1 Hz) and spatially smoothed with a Gaussian kernel (full-width at half maximum = 6 mm).

### Functional Stability Calculation

The calculation of functional stability was performed according to methods used in recent studies (18, 24) and implemented by the DPABI toolbox. We used a sliding-window approach to carry out a dynamic functional connectivity analysis, with a window size of 64 s and a sliding step of 4 s (18, 24). As the parameter setting in the sliding-window approach remains controversial (15), the additional following procedures were performed to further verify our results. First, the analyses were performed with three different window lengths (=35, 50, and 80 s) to examine whether the results were influenced by the choice of window length. Second, the analyses were conducted with different sliding steps (=2 s) to identify whether the results were dependent on the selection of different sliding steps.

For a given voxel j, the Pearson's correlation coefficients between its time course and those of all other voxels within the gray matter mask were calculated. This yielded a series of dynamic functional connectivity maps across time windows for voxel j. The functional stability of voxel j was subsequently quantified with the Kendall's concordance coefficient (KCC) of these dynamic functional connectivity maps using time windows as raters. KCC is calculated with the following equation:


$$\text{KCC=}\frac{\sum_{\text{n=1}}^{\text{N}}{\text{R}}_{\text{n}}^{\text{2}}-\frac{\text{1}}{\text{N}}-{\left(\sum_{\text{n=1}}^{\text{N}}{\text{R}}_{\text{n}}\right)}^{\text{2}}}{\frac{\text{1}}{\text{12}}{\text{K}}^{\text{2}}\left({\text{N}}^{\text{3}}-\text{N}\;\right)},$$

where K is the number of time windows, N is the number of connections between voxel j and all other voxels within the gray matter mask, and Rn is the sum of rank for the *n*-th connection across all windows. The connections for each window are ranked across all voxels based on their functional connectivity strength. The gray matter mask that was employed to confine the analyses in this work was generated by thresholding the mean gray matter density map across participants at 0.2. After deriving the functional stability maps, they were further standardized into *z*-scores by subtracting the mean and dividing by the standard deviation of global values within the gray matter mask. They could then be averaged and compared across subjects. A higher stability value (KCC) for a voxel or a region indicates that its dynamic functional architecture configuration remains more consistent and stable over time, while a lower stability value is indicative of its ability to frequently and rapidly shift between one brain state and another.

### Statistical Analysis

The between-group comparison of demographic variables such as age and education level was performed using the non-parametric Mann–Whitney *U*-test, and the chi-square test was used to compare the categorical variables (e.g., sex); the statistical significance was set at *P* < 0.05.

For each group, the one-sample *t-*test was conducted to investigate the profile of intrinsic functional stability across the brain. The two-sample *t*-test was used to assess the difference in functional stability between the two groups in a voxel-wise manner. The statistical threshold was set at *P* < 0.05 corrected by the Gaussian Random Field (GRF) method (with the voxel-level *P* < 0.001). Following the two-sample *t*-test, the areas that exhibited significant differences in functional stability were regarded as the regions of interest (ROIs) and the mean values of functional stability in these ROIs were calculated. Subsequently, Spearman's correlation analyses were used to evaluate the relationship between the functional stability index and ALSFRS-R among the ALS patients; a *P* < 0.05 was regarded as statistically significant.

## Results

**Figure 1:**
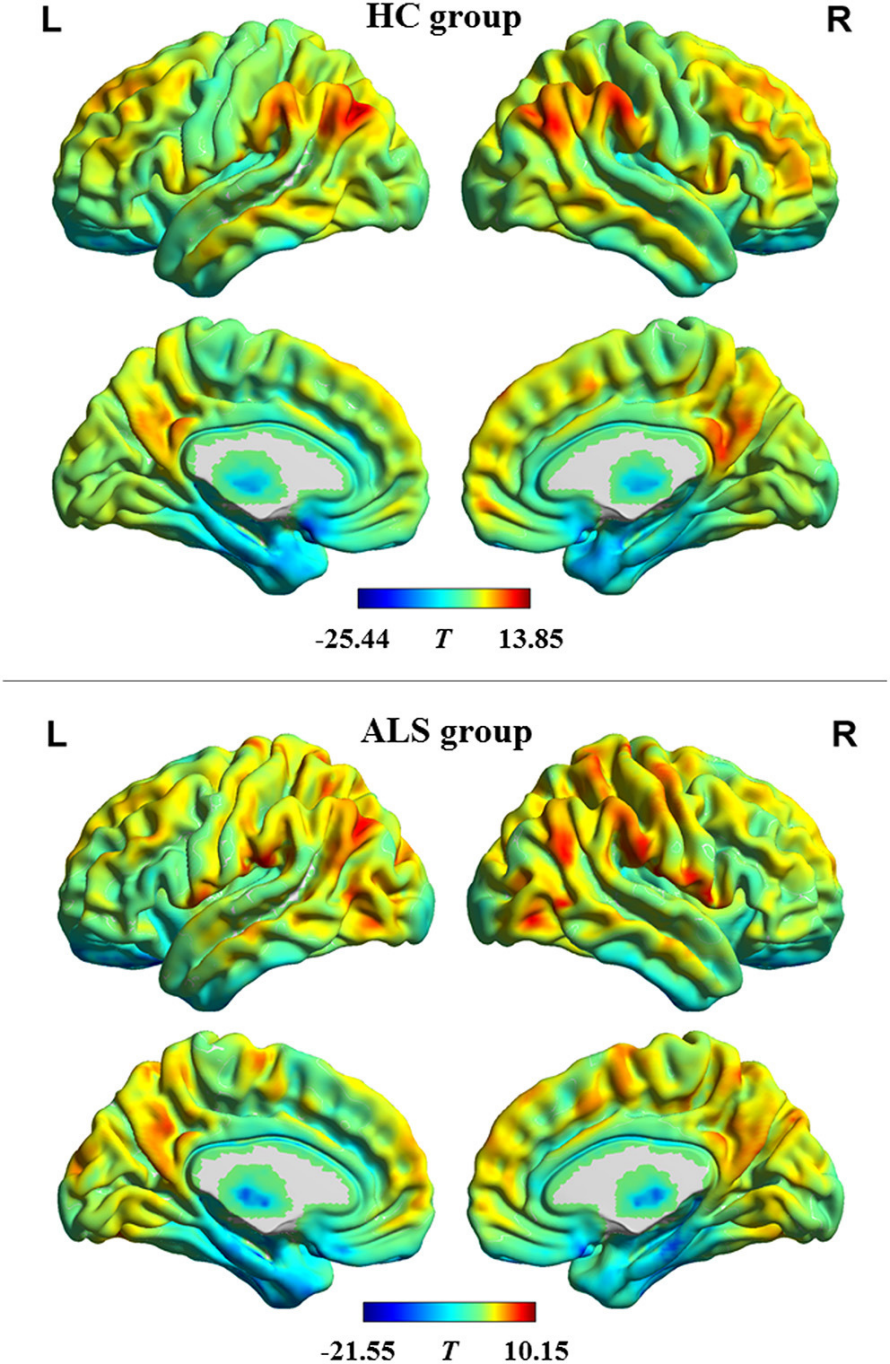
The profile of intrinsic functional stability within each group. The results of one-sample *t-*tests on functional stability in the resting-state are shown in brain maps of *T*-values. Red and blue indicate high and low stability, respectively. HC, healthy control; ALS, amyotrophic lateral sclerosis; L, left; R, right.

The profiles of intrinsic functional stability within each group are shown in Figure 1. By visual inspection, a set of brain areas with relatively higher values of functional stability were bilaterally observed in the HC group. These areas mainly involved the lateral pre-frontal cortex, superior and inferior parietal lobule, posterior cingulate cortex and precuneus, medial pre-frontal cortex, occipital cortex, and lateral temporal cortex. In contrast, relatively lower functional stability was observed in several brain areas, including the orbitofrontal cortex, pre-central and post-central cortex, paracentral lobule, temporal pole, medial temporal cortex, and subcortical nucleus. This profile of intrinsic functional stability was consistent with previous reports (18, 24). Additionally, the ALS group exhibited a similar spatial distribution of functional stability.

**Figure 2:**
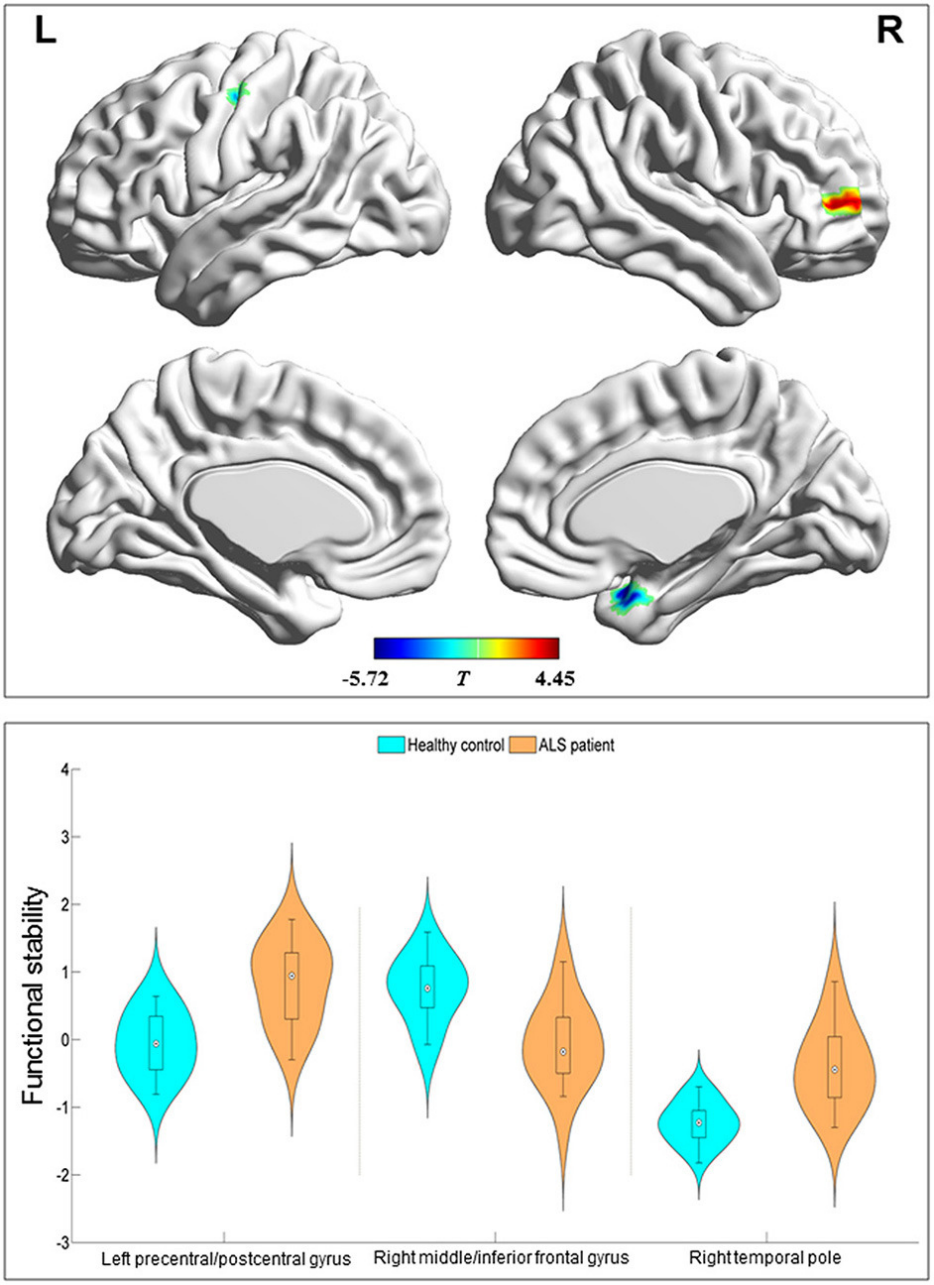
Regions with between-group differences in functional stability. The red and blue color indicate the regions with the decreased and increased functional stability in the patients with amyotrophic lateral sclerosis, respectively. The distribution and between-group differences are shown in the violin and box plots (all *P* < 0.0001) of mean value of functional stability in these regions. L, left; R, right.

Compared with the HC group, the ALS group had significantly increased functional stability in the left pre-central and post-central gyrus and right temporal pole and showed significantly decreased functional stability in the right middle and inferior frontal gyrus (Figure 2 and Table 2). The analyses performed using different sliding-window parameter settings revealed very similar patterns of between-group difference in functional stability, thereby verifying the robustness of the results (Supplementary Figure 1).

**Table 2:** Regions with between-group difference in functional stability.

**Regions**	**Voxels**	**Brodmann area**	**MNI coordinates**			**Peak *T* value**
			**x**	**y**	**z**	
Left pre-central and post-central gyrus	45	3/4	−45	−18	48	−5.72
Right middle and inferior frontal gyrus	33	10	39	54	6	4.45
Right temporal pole	31	38	36	9	−24	−5.03

**Figure 3:**
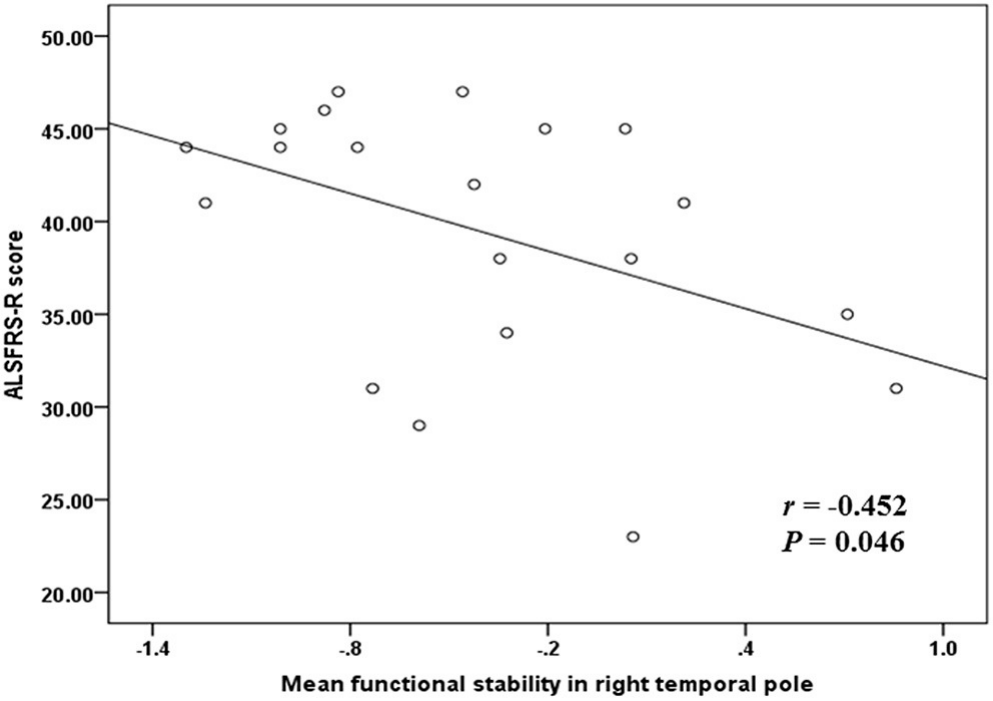
The correlation between functional stability and disease severity reflected by ALSFRS-R score. ALSFRS-R, revised ALS Functional Rating Scale.

Correlation analyses were performed and indicated a significant negative correlation (*r* = −0.452 and *P* = 0.046) between the mean functional stability in the right temporal pole and the ALSFRS-R score in the ALS group (Figure 3).

## Discussion

To our knowledge, our study details the first use of resting-state fMRI to perform functional stability analysis in ALS. The main findings were as follows. (1) The within-group analysis indicated that higher functional stability was primarily distributed in the default mode network and the frontal parietal network, while lower functional stability was mainly distributed in the SMN, auditory network, and limbic network. For the most part, this profile was in accordance with the stability pattern found in previous studies (18, 24, 27, 28). (2) ALS patients exhibited altered functional stability characteristics during the resting state. The sensorimotor region, including the left pre-central and post-central gyrus, had increased functional stability, while the non-sensorimotor areas, including the right temporal pole and the right middle and inferior frontal gyrus, had increased and decreased functional stability, respectively, in ALS. These findings indicate that ALS is a neurodegenerative disease that affects multi-systems. (3) The disease severity was significantly correlated with the mean functional stability in the right temporal pole, which suggested the potential of functional stability as an indicator of disease progression.

Our findings of abnormal stability of dynamic FC in ALS can be supported by studies related to neurophysiology. Resting-state EEG studies have found the dominance of slower EEG frequencies in their oscillatory activity (29) and increased global gamma power in ALS (30). Additionally, resting-state EEG has revealed that ALS patients have an increment in the intra-motor cortical FC (31). Furthermore, EEG-related studies have demonstrated alteration in cortical networks, such as the frontoparietal network, frontotemporal network, and sensorimotor network, in ALS (32, 33). It is worth noting that simultaneous resting-state EEG-fMRI studies have revealed a link between dynamic FC in fMRI data and concurrent EEG signals (34, 35). Because functional stability represents the coherence of dynamic FC over time, the above EEG studies may suggest the changes in functional stability in ALS that can be expected.

The patients with ALS exhibited higher functional stability in the left pre-central and post-central gyrus, which is responsible for somatosensory and motor functions. The increase of functional stability may indicate the limited ability of these areas to quickly transfer from one brain state to another (18). In accordance with our findings, several studies from a dynamic perspective have found a reduction in ReHo (regional homogeneity, a functional index reflecting the regional coherence of brain activity) in bilateral sensorimotor cortices in ALS (36, 37). From the perspective of static FC, previous studies have reported decreased connectivity in SMN (9, 10, 38, 39); moreover, from a dynamic point of view, a recent FC study based on resting-state fMRI also found that SMN was involved in ALS (13). These reports in static and dynamic FC studies are in keeping with our findings. Given that somatosensory and motor dysfunctions are often observed in ALS (3), it was implied that the altered functional stability in the sensorimotor region may be one of the underlying pathophysiological mechanisms of sensory and motor dysfunction in ALS.

ALS patients showed a higher stability of FC in the right temporal pole, indicating that this region may be one of the pathological nodes of ALS. In agreement with our results, it has been revealed that the perfusion of bilateral temporal poles is decreased in ALS (40). In addition, the previous study demonstrated the correlation between the altered metabolism of the right temporal pole and cognitive dysfunction in ALS (41). The temporal pole is thought to be a node of the paralimbic cortex that plays a key role in language and memory functions (42, 43). Abnormal functional stability in this region might lead to the deficits in semantic processing and memory, all of which are observed in ALS (44).

In addition, the right middle and inferior frontal gyrus [Broadmann area (BA) 10] were found to show decreased functional stability in ALS in this study. The reduction of stability may lead to a lower ability to maintain the consistent functional coordination of these frontal areas with other brain regions (18). Consistent with our findings, a positron emission tomography study found pre-frontal (BA 9 and 10) dysfunction (reflected by glucose hypometabolism) in ALS (45). In addition, an fMRI study observed decreased FC in frontal areas including the middle and inferior frontal gyrus (46). The pre-frontal cortex is an important component of the neural circuitry that processes top-down behavioral control and functions in cognitive control (47). Thereby, it is implied that the decreased functional stability in the right middle and inferior frontal gyrus might lead to cognitive (such as attention and executive function) and behavioral impairments, all of which are often observed in ALS (48).

Our work herein has several limitations. The sample size was relatively small, which may have limited the generalizability of the findings. To better explore the subtle brain abnormalities in ALS, further studies are needed to consider larger samples to provide sufficient statistical power. Second, to better explore the biological significance of altered functional stability in ALS, future works are recommended to combine electrophysiological methods such as EEG and resting-state fMRI. Third, we adopted the sliding window method to extract dynamic FC, which is widely used in current research. To avoid potential bias, future research can consider using other extraction approaches [e.g., the point-process method (49)] to analyze dynamic FC in ALS. Forth, the current study did not conduct cognitive assessment in ALS patients; thereby, the speculation about the relationship between cognitive dysfunction and the altered functional stability should be further verified in the future study.

In summary, our observations revealed that the abnormal pattern of FC stability involved sensorimotor regions (i.e., the left pre-central and post-central gyrus) and non-sensorimotor areas (i.e., the right temporal pole and middle/inferior frontal gyrus) in ALS patients. These results provide additional evidence to support the idea that ALS is a multi-system disorder. Our preliminary findings also suggest the possibility that functional stability may serve as a biomarker for monitoring the progression of ALS.

## Data Availability Statement

The original contributions presented in the study are included in the article/Supplementary Material, further inquiries can be directed to the corresponding author.

## Ethics Statement

The studies involving human participants were reviewed and approved by the Research Ethics Committee of Fujian Medical University Union Hospital. The patients/participants provided their written informed consent to participate in this study.

## Author Contributions

JW: data curation, formal analysis, investigation, and writing—original draft. J-HL: formal analysis, investigation, and visualization. L-MC: formal analysis, investigation, validation, visualization, and writing—original draft. J-YS and X-HZ: data curation, investigation, and writing—original draft. Z-YZ: formal analysis, funding acquisition, and investigation. H-JC: conceptualization, data curation, formal analysis, funding acquisition, investigation, project administration, supervision, visualization, and writing—review and editing. All authors contributed to the article and approved the submitted version.

## Funding

This work was supported by the National Natural Science Foundation of China (Grant nos. 82071900, 81671271, and 81974199), Fujian Province Natural Science Foundation (Grant no. 2021J01759), and Fujian Province Joint Funds for the Innovation of Science and Technology (Grant no. 2019Y9067).

## Conflict of Interest

The authors declare that the research was conducted in the absence of any commercial or financial relationships that could be construed as a potential conflict of interest.

## Publisher's Note

All claims expressed in this article are solely those of the authors and do not necessarily represent those of their affiliated organizations, or those of the publisher, the editors and the reviewers. Any product that may be evaluated in this article, or claim that may be made by its manufacturer, is not guaranteed or endorsed by the publisher.

## Supplementary Material

Supplementary Figure 1 The effect of different sliding-window parameter settings on functional stability analysis. The sliding-window approach was used for the dynamic functional connectivity analysis, with the following parameter settings: **(A)** window size = 35 s and sliding step = 4 s; **(B)** window size = 50 s and sliding step = 4 s; **(C)** window size = 80 s and sliding step = 4 s; **(D)** window size = 64 s and sliding step =2 s. The main findings of the between-group comparison could be reproduced by performing analyses of functional stability based on different window lengths and sliding steps, suggesting that different sliding-window parameter settings did not significantly influence the results.

## Abbreviations

ALS: amyotrophic lateral sclerosis
fMRI: functional magnetic resonance imaging
FC: functional connectivity
SMN: sensorimotor network
ALSFRS-R: revised ALS Functional Rating
FD: frame-wise displacement
KCC: Kendall consistency coefficient
EEG: electroencephalography.
