# Supplementary figures and images for: Abnormal Stability of Dynamic Functional Architecture in Amyotrophic Lateral Sclerosis: A Preliminary Resting-State fMRI Study
Source: Front Neurol. 2021 Oct 13;12:744688. doi: 10.3389/fneur.2021.744688 (PMC8548741; doi:10.3389/fneur.2021.744688)

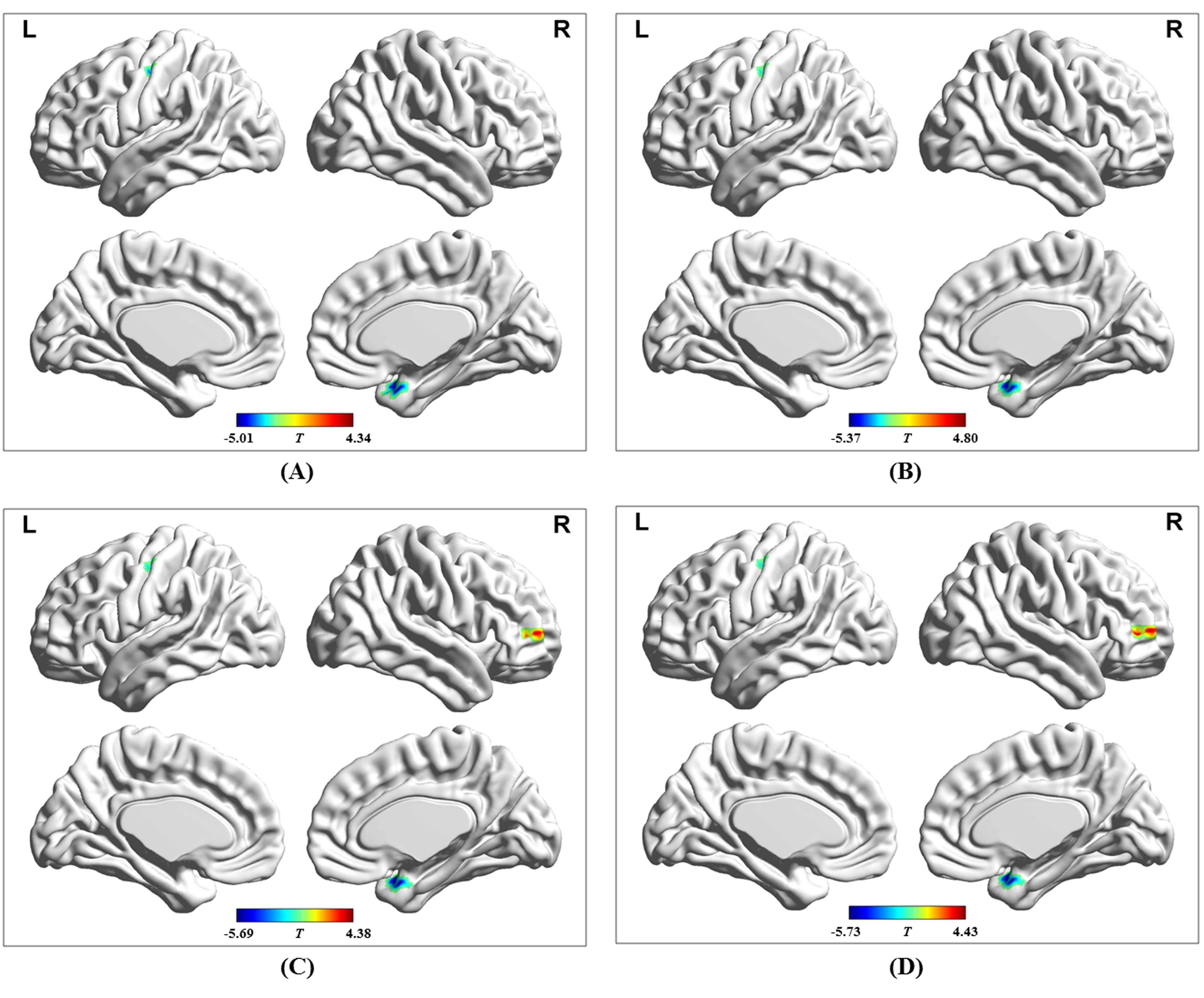

Supplement: Supplementary Figure 1 — The effect of different sliding-window parameter settings on functional stability analysis. The sliding-window approach was used for the dynamic functional connectivity analysis, with the following parameter settings: (A) window size = 35 s and sliding step = 4 s; (B) window size = 50 s and sliding step = 4 s; (C) window size = 80 s and sliding step = 4 s; (D) window size = 64 s and sliding step =2 s. The main findings of the between-group comparison could be reproduced by performing analyses of functional stability based on different window lengths and sliding steps, suggesting that different sliding-window parameter settings did not significantly influence the results. [file Image_1.TIF]
